# Exploring implementation of a shared decision-making intervention for patients following an Anterior Cruciate Ligament rupture: a qualitative investigation

**DOI:** 10.1186/s12911-026-03430-3

**Published:** 2026-03-17

**Authors:** Hayley M. Carter, David J. Beard, Charlotte Dodsley, Paul Leighton, Joshua McCallion, Fiona Moffatt, Benjamin E. Smith, Kate E. Webster, Pip Logan

**Affiliations:** 1 Physiotherapy Outpatients, Level 3, Florence Nightingale Community Hospital, Derby, DE1 2QY UK; 2 School of Medicine, University of Nottingham, Queens Medical Centre, Nottingham, NG7 2UH UK; 3 Surgical Intervention Trials Unit, Botnar Research Centre, NDORMS, University of Oxford, Windmill Road, Oxford, OX3 7LD UK; 4 NHMRC Clinical Trials Centre, Faculty of Medicine and Health, University of Sydney, Syndey, Australia; 5 Patient representative, The POP-ACLR Study, Derby, UK; 6 School of Health Sciences, University of Nottingham, Queen’s Medical Centre, Nottingham, NG7 2HA UK; 7 School of Allied Health, Human Services and Sport, La Trobe University, Kingsbury Drive, Bundoora, VIC 3086 Australia; 8 Surgical, Treatment and Rehabilitation Service (STARS) Education and Research Alliance, University of Queensland, Herston, 4006 Australia

**Keywords:** Anterior cruciate ligament, Extended normalisation process theory, Implementation factors, Feasibility study, Shared decision-making

## Abstract

**Background:**

Anterior Cruciate Ligament (ACL) ruptures affect over 20,000 individuals in the UK each year. To date, three randomised controlled trials have compared the two main treatment options of surgical and non-surgical management following rupture and report conflicting findings. Recent qualitative research has also reported uncertainty and confusion among patients regarding decision-making about their choice of treatment. A theory- and evidence-based shared decision-making intervention was co-developed in a prior study to support patients to decide on treatment. The aim of this study was to understand implementation factors associated with implementing a shared decision-making intervention for patients following an anterior cruciate ligament (ACL) rupture.

**Methods:**

Individual qualitative interviews, analysed using a framework approach underpinned by the Extended Normalisation Process Theory (ENPT). Data were mapped to the four ENPT constructs: potential, capacity, capability and contribution. Conducted as part of a non-randomised feasibility study in an orthopaedic and physiotherapy service at an acute National Health Service Teaching Hospital in the Midlands, UK. Five patients with a first time ACL rupture and five physiotherapists with experience of using the shared decision-making intervention.

**Results:**

Implementation factors, including barriers and enablers to future implementation and normalisation, associated with implementing a shared decision-making intervention for patients following ACL rupture have been identified and mapped to ENPT. Both patients and physiotherapists demonstrated a clear understanding of the SDM intervention’s purpose (coherence) and individual intention to operationalise it within the pathway (potential, capacity). The intervention was further discussed to support interactional work between patients and clinicians during consultations and physiotherapists were positive about its workability and adaptability to meet patients’ needs (capability). Physiotherapists described the intervention’s role in altering the social roles of both themselves and of patients, which supported the set-up and action of SDM conversations (capacity). The communication of equipoise and context created for intervention delivery was identified to be critical to engagement (potential). The intervention was described as non-burdensome, patient participants reported they would recommend it to others following diagnosis of an ACL rupture and physiotherapist participants described utilising the intervention with patients outside the trial (contribution, capability).

**Conclusion:**

Implementation factors, explored through the lens of ENPT, have supported understanding of future normalisation of the intervention in clinical practice. Areas of focus ahead of further research, for example understanding equipoise, have also been identified.

**Trial registration:**

ISRCTN17801081. Registration date: 23.01.2024.

**Supplementary Information:**

The online version contains supplementary material available at 10.1186/s12911-026-03430-3.

## Background

Decision-making about treatment following an Anterior Cruciate Ligament (ACL) rupture can be challenging [1], due to the disparity in results from three randomised controlled trials (RCTs), comparing surgical (ACL Reconstruction, ACLR) and non-surgical treatment (rehabilitation). A 2010 RCT (KANON study) concluded that ACLR may not be appropriate for all, and a trial of rehabilitation first may prevent unnecessary surgical treatment for up to 50% of patients [2]. In contrast, a 2021 RCT (COMPARE trial) comparing early ACLR with rehabilitation and the option of delayed ACLR, reported improved outcomes in favour of early surgery at 2-year follow-up [3]. Most recently, a 2022 RCT (ACL SNNAP trial) concluded superior outcomes for ACLR at 18-months when compared with non-operative treatment for patients with non-acute ACL injuries [4].

A shared decision-making (SDM) intervention was developed with patients and key stakeholders to support the decision to proceed with surgical (ACLR) or non-surgical management (rehabilitation) [5]. The SDM intervention was explored in a feasibility study with embedded qualitative interviews with patients and physiotherapists at one large acute NHS teaching hospital [6]. The study demonstrated that it was feasible and acceptable to deliver the SDM intervention in an NHS setting with indicators of effectiveness also identified, and reported separately [7]. A secondary aim of the feasibility study was to explore implementation factors to support future research and translation of the intervention into clinical practice, which is presented in this paper.

The Extended Normalisation Process Theory (ENPT) underpinned the study and supported evaluation of implementation. ENPT has previously been identified as a valuable tool to support intervention implementation for orthopaedic and musculoskeletal conditions [8]. ENPT was described by May in 2013, expanded from the original version of the theory (NPT), to consider dynamic implementation contexts in which an intervention operates [9]. Four constructs were defined to include: (1) potential, (2) capacity, (3) capability and (4) contribution. Potential relates to an agents’ individual attitudes and intentions and, shared commitment and values to operationalise the intervention. Capacity denotes the dynamic elements of context, considering social norms and roles, material and cognitive resources available to agents (within a system) to operationalise the intervention. Capability describes factors related to the workability and integration of an intervention within usual practice. Finally, contribution considers an agent’s continuous contributions to enact the intervention including meaning and sense-making (coherence), commitment and engagement (cognitive participation), skills and resources used (collective action) and translation of the interventions appraisal to reconfigure action (reflexive monitoring).

This paper reports the results of the embedded qualitative interviews with specific focus on implementation factors.

## Methods

Individual qualitative interviews were embedded within a non-randomised feasibility study. Results pertaining to feasibility and acceptability are explored in a separate publication.

To support implementation of the intervention in the trial, the Implementation Research Logic Model (IRLM) was used [10]. Implementation strategies were mapped to ENPT [9]. The IRLM is available in supplementary file 1.

This study was approved by East Midlands - Nottingham 1 Research Ethics Committee (REC reference: 23/EM/0263). All those who participated in an interview provided informed consent. This study was reported in accordance with the Consolidated criteria for reporting qualitative research (COREQ) checklist [11], which is available in supplementary file 2.

### Sampling and recruitment

Participants were recruited between 29 January 2024 and 5 June 2024 from the orthopaedic and/or outpatient musculoskeletal physiotherapy services at one large acute NHS teaching hospital, across three sites. On consenting to take part in the feasibility trial, participants were also asked to provide consent to be later contacted to take part in an individual interview exploring their experiences of being involved in the trial. Eligibility criteria for participants in the feasibility trial is shown in Table 1.

**Table 1:** – Eligibility criteria

**Inclusion criteria**
• Age 18 or over • First-time ACL rupture (in that limb) confirmed by a magnetic resonance imaging (MRI) scan
**Exclusion Criteria**
• Concomitant injury requiring surgical intervention anticipated to significantly alter usual treatment e.g. fracture, bucket handle meniscal tear requiring immediate surgical intervention prior to ACLR • Previous surgery to the affected limb • Pregnancy (as this is likely to affect decision-making regarding surgical treatment and rehabilitation)

Those providing consent were purposively sampled to include variation of age, sex, ethnicity, deprivation data (according to the Indices of Multiple Deprivation 2019 (IMD2019) Decile which represents ten equal sized groups from one (most deprived 10% of areas nationally) to ten (least deprived 10% of areas nationally) [12]), risk of poor health literacy as assessed by the Rapid Estimate of Adult Literacy in Medicine, Revised (REALM-R) [13], level of education and, responses to follow-up questionnaires. Two follow-up questionnaires were collected as part of the wider feasibility trial: (1) a bespoke questionnaire including 15 statements pertaining to key elements thought to determine acceptability of the SDM intervention (supplementary file 3) and (2) the Satisfaction with Decision (SWD) scale which comprises of six items scored on a five-point Likert scale to determine a patient’s satisfaction with a healthcare decision [14, 15]. For each item a level of satisfaction is reported from strongly agree (5) to strongly disagree (1) with an overall level of satisfaction reported between 5 (low satisfaction with decision) to 30 (high satisfaction with decision). The SWD scale is shown in supplementary file 4.

Physiotherapists delivering the intervention were identified at the end of the trial, were purposively sampled and invited to interview. They were provided with the study information sheet and consent form.

### Data collection

All semi-structured interviews were conducted using a topic guide (available in supplementary file 5) by HC, a female Physiotherapist, PhD candidate at the University of Nottingham and chief investigator of the feasibility study. The interviewer had no previous relationship with the patient participants but has worked with some physiotherapists in the study as a clinical colleague.

Participants were given the option of a virtual (telephone or Microsoft Teams) or in-person interview. Interview length ranged from 17 to 47 minutes.

### Data analysis

The framework approach was used to analyse qualitative data which was mapped to the four constructs of ENPT: (1) Potential, (2) Capacity, (3) Capability and (4) Contribution. Contribution was further divided into four dimensions (mirroring the structure of ENPT): (a) coherence. (b) cognitive participation, (c) collective action and (d) reflexive monitoring.

Interviews were recorded and transcribed verbatim using an approved third-party transcription service (TP transcription, https://tptranscription.co.uk). All transcriptions were checked for accuracy against original recordings. A reflexive journal was maintained by the interviewer to document thoughts after each interview, on initial reading of the transcripts and during data analysis.

Data were discussed with the study team during trial management meetings after charting was completed for 25% of the dataset. Following mapping, data were organised into two broad topics. Topic 1: acceptability, indicators of effectiveness and feasibility. Topic 2: implementation of the intervention and considerations for future implementation and normalisation. Topic 2 is explored in this manuscript, topic 1 is explored in a separate publication [7].

### Patient and public involvement

Patients and stakeholders (such as therapists, orthopaedic surgeons and therapy managers) with experience of having, treating or managing departments treating ACL injuries were involved in several aspects of the research from design to dissemination. PPI activities supported key decision-making about study design (such as participant eligibility criteria, sample size, patient facing documentation), methods of inquiry, data analysis and dissemination strategies.

## Results

Of the 20 patient participants in the feasibility study, 15 consented to be contacted for an interview and five were interviewed. The median age of participants was 32 (range 20–57), two were female, one identified as Asian Pakistani and four identified as white British. Participants level of education ranged from level 2 to 6, and all scored above 6 on the REALM-R (indicating a low risk of poor health literacy). Participants included those who injured their ACL via a contact injury and those with a non-contact mechanism, with a median time between injury and diagnosis of 2-months (range 5 days to 6 years). A range of physical activity levels and frequencies were reported prior to injury (lowest: recreational activity 2–4 times-a-week, highest: competitive national level competition 5–7 times-a-week). Employment status included three participants working full-time, one working part-time and one student.

In addition, five physiotherapists involved in intervention delivery were purposively sampled to be interviewed. They had a range of years’ experience as qualified physiotherapists at Agenda for Change Bands 5–7 (Band 5 – newly qualified/junior, Band 6 – senior, Band 7 – specialist) and treated a varying number of patients participating in the trial (range: 1–3) at either site with different appointment lengths (30–60 min) and mediums (telephone call and face-to-face).

Interview data sought to explore how the intervention was operationalised and understand considerations for future implementation in practice. The data is described under four themes mapped to each construct of ENPT: potential (theme 1), capacity (theme 2), capability (theme 3) and contribution (theme 4).

### Theme 1: Potential

Implementation of the SDM intervention depends upon participants’ commitment to operationalise it through individual intention and shared commitment. Both patients and physiotherapists demonstrated individual intention to operationalise the intervention. Patients discussed their beliefs and attitudes towards being involved in decision-making about their care, with some reporting conflict between their personal values and social norms and roles. For example, one patient responded to ‘neither agree nor disagree’ to the following statement on the SWD scale ‘the decision I made was the best decision possible for me’. When explored during interview, they described that despite their motivation to engage in SDM, they were unsure of their potential to do so as they didn’t feel involved in the decision.

*I was still in the frame of mind that the decision perhaps wasn’t really in my hands*,* as such. I guess having a consultant say to you*,* “You are having surgery*,*” or*,* “I’m listing you for surgery*,*” you kind of trust their judgement. … I think at the time of filling that out I was probably a little bit more on the fence about whether I was able to make the decisions on my own. [P0015*,* patient]*

They explained that they were continually revisiting the SDM intervention and each time gained a greater understanding of their injury, the treatment options available to them and questions they wished to discuss at upcoming appointments. The intervention subsequently supported them to enact upon their beliefs and behaviours to be involved in treatment decision-making and challenge the system norms and roles they had previously experienced.

Physiotherapists also displayed individual intention to enact the SDM intervention. They described their beliefs to offer patients informed choice by presenting evidence-based information to support all available treatment options. Physiotherapists recognised the role of the intervention in operationalising this belief (coherence) which also fit with their perception of the role of an initial assessment and the work they deliver. The SDM intervention was described to align to the process and structure of an initial assessment, demonstrating congruence between the intervention and the system in which it operates. Physiotherapists expressed motivation to enact the SDM intervention with all patients and discussed its utility both within and outside of the trial setting. All physiotherapists discussed their desire to tailor a consultation to meet individual patient needs and recognised the intervention as adaptable to achieve this goal (capability).

Physiotherapists further discussed the importance of the language used to communicate treatment options (and convey equipoise) and the impact this had on enacting the SDM intervention. They shared their experiences of treating patients who had been advised an ACLR was necessary and how they found it difficult to consider alternative treatment options that seemed to contradict the surgeon’s recommendation, subsequently making it challenging to engage with SDM. Physiotherapists reflected on the need for a shared commitment to provide an appropriate context for the delivery of and for patients to engage with SDM.

*I think it would be really useful [to use with patients following diagnosis of an ACL rupture]. I think it should be done at first point of access. Whoever’s giving the diagnosis of ACL rupture … this patient*,* he’s already been told he was having a reconstruction*,* so it was already too late. I think it would be helpful to do [shared decision-making] at diagnosis … because then you’re getting it [the intervention] at the right time and it’s an informed choice then*,* at that point. [P0026*,* clinician]*

Physiotherapists felt that the responsibility for creating and sustaining shared commitment was owned by the orthopaedic team, primarily medical colleagues, such as consultants and registrars, who would typically be consulting with patients at the point of diagnosis. This shared commitment across the pathway builds collective readiness to both enact the SDM intervention and respond to changes brought about by its operationalisation. Where patients reported that they had been advised that they needed an ACLR, and consequently felt as though they had less influence over the decision about treatment, they still reflected on the intervention’s utility in supporting improvements in their knowledge, demonstrating individual commitment in different clinical scenarios.

### Theme 2: Capacity

Implementation of the SDM intervention depends on the capacity of participants to coordinate their actions. As a result, implementation modifies clinical practice and its possible outcomes and impacts upon the social roles, norms and conventions that govern the conduct of participants.

Both patients and physiotherapists demonstrated intent to incorporate the intervention within the care pathway deliberately. Physiotherapists made investment in building co-operative capacity and discussed deliberate actions to deliver the intervention during their initial appointments. Capacity was supported by: (1) delivery of the trial intervention training, (2) use of the fidelity checklist outlining essential components to be delivered during the consultation (although developed to measure fidelity for completion after the initial appointment, some physiotherapists described it’s use during the appointment as a ‘checklist’), (3) trial summary information received via email and paper copies available in the department, (4) trial website and (5) a responsive contact for a research team member. A culture of cooperation was further developed as physiotherapists communicated with each other about their experiences in the trial, offering peer support to deliver the intervention.

However, a key problem discussed by physiotherapists was certainty of engagement with the intervention during prior consultations in the patient’s journey. They discussed dealing with this uncertainty and its impact on their ability to operationalise the intervention and engage in what they viewed as an SDM process.

*People would be less open to have the discussion*,* if they’ve already been told in another consultation that repair is the best option for them. They’ll obviously be closed off to what you’re trying to say because a consultant with more expertise has said otherwise…. it would be good to have this [the intervention] at every level*,* so be done at the initial appointment*,* initial consultation with the surgeon who I guess is the one listing them at that time. Because … there must have been at least one if not two that had been put on the waiting list for the repair. So I guess paid less attention because they’d already made that decision with the consultant … I don’t know what discussions were had at that stage*,* but it would be good to know that a similar level of conversation or education was happening at each level of their journey*,* I guess*,* so that you’re singing from the same hymn sheet. [P0022*,* physiotherapist]*

Physiotherapists also discussed the role of the intervention in altering the social roles of both themselves and the patients within the ACL rupture pathway. They reflected that, in comparison with treatment of non-trial patients receiving usual practice (no SDM intervention), trial patients were expectant of involvement in a SDM conversation during their consultation. This was also corroborated by patients who responded positively to receiving an intervention intended to support them to make treatment decisions, widening the resources available to them.

In contrast, one participant appeared to struggle with their involvement in decision-making and the change to their social norms and roles. They explained that they had been told by their orthopaedic consultant that they needed surgery, but after engaging with the SDM intervention felt less confident ACLR was essential. They reflected on their experience in dealing with this uncertainty and difficulty acknowledging their new role in contributing to decision-making about their care. They explained that the intervention offered new resources which supported them to understand their options, digest information away from the clinical consultation and formulate questions to discuss with healthcare professionals. Whilst they reported the intervention helped them to come to a decision, they also stated that they were undecided about treatment and so had referred back to the decision made by the surgeon; demonstrating ongoing difficulty with responsibility for treatment decision-making.

*When I mentioned that I wanted to return to running*,* he [orthopaedic consultant] just said straight away*,* knee surgery*,* and I was told that if I didn’t have the knee surgery it would always let me down and possibly do more damage. So he was quite clear cut and dried … then you came in and was able to give me all the more information [the intervention] and that I found … really helpful because we [patient and their partner] then had time to have a look at it. Unfortunately … the decision wasn’t really obvious. … But your leaflet I found really helpful and that’s a good thing to give out to people like me because there doesn’t seem to be time in the appointments to digest what you’ve been told and formulate sensible questions. … It sort of helped me to come to a decision. The decision I think has been based on the fact that the surgeon just said*,* “Oh you need surgery*,* it’s always going to let you down” and he was quite certain about it and I just had to sort of think*,* well we’ve seen a few of these so that’s what I’m going to have to go with. You see I’m still not dead sure*,* it is difficult [P0009*,* patient]*

Further, one participant expressed a perceived lack of intent from their physiotherapist to enact the SDM intervention. They felt their physiotherapist’s priority was to assess levels of pain, range of movement and provide exercises and due to time pressures, had less availability to focus on the SDM conversation.

*I do wish the physio showed a little bit more intent*,* but I understand*,* you know*,* how busy they are and how understaffed they are in certain areas and I appreciate that their job’s so difficult … but just maybe a little bit more empathy and a little bit more showing of understanding might have been useful. [P0012*,* patient]*

### Theme 3: capability

The potential of participants to operationalise the intervention depends on its workability and integration with usual practice.

The SDM intervention was discussed to support interactional work between patients and clinicians who used it to structure conversations, ask and answer questions. Physiotherapists reported the intervention to flow with their usual practice during an initial consultation held in-person or via telephone lasting 30 or 60-minutes.

*“from how I work education is a huge part of that*,* to make sure a patient understands surgically what’s happened*,* what they’ve done*,* or if they’ve had images taken what they show*,* and explain and make sure they’ve got an understanding of that before you leap into treatment. So this facilitated that and works in a similar way that the patient is then well informed before you then start training*,* so there’s better buy-in*,* better engagement. So it aligned quite nicely with how I work.” [P0022*,* physiotherapist]*

Physiotherapists were positive about the workability of the intervention suggesting it to be adaptable to meet patient needs. Integration was supported by use of the intervention fidelity checklist, trial summary emails received ahead of a patient consultation and through use of a paper copy of the intervention during face-to-face appointments. Although all physiotherapists felt able to enact the intervention and agreed it was compatible with their skill set, one physiotherapist raised concerns about the capability of more junior physiotherapists with fewer years’ experience to deliver the intervention.

*it probably depends on the level of expertise*,* perhaps. If you’re a Band 5 rotational*,* would you have the confidence to do it [deliver the SDM intervention]? Maybe not [P0026*,* physiotherapist]*

However, an interview conducted with a Band 5 (junior) physiotherapist did not support this, and instead suggested the intervention supported their confidence to deliver SDM.

The capability to operationalise the intervention outside of an NHS setting was also demonstrated as patient participants reported enacting it with friends, family members and other healthcare professionals (namely physiotherapists).

*So I took the material with me to [the university] Physio and they’ve seen it all. … For me the study was really useful and the physio had a look through it as well and*,* yes*,* we both were kind of on the same page. [P0012*,* patient]*

### Theme 4a: contribution (coherence)

All physiotherapists participating in the trial demonstrated a clear understanding of the intervention’s purpose and recognised the role of the trial training in supporting this. They viewed the intervention as novel and suggested that it was more effective than usual care (discussed above). There was further evidence of patients and physiotherapists working collaboratively to build a shared understanding of the intervention’s role and potential benefits of its use. Patients further made sense of the intervention in comparison to other resources and described its distinct differences in offering greater detail about the injury and treatment options, collating research in one accessible document, using appropriate medical terminology, presenting information with a mix of text and figures, displaying references clearly, provoking thought to consider relevant questions and offering a platform to welcome discussion about treatment options.

*the NHS website’s fine but this goes into a lot more detail … you always think you know what a certain injury is but actually what does it truly involve? And this document has really helped me just digest it all. [P0015*,* patient]*

### Theme 4b: contribution (cognitive participation)

The commitment to enacting the intervention has been discussed above with both patients and clinicians demonstrating individual intentions to operationalise it (potential). Physiotherapists suggested the SDM intervention provided a framework for discussion with patients and discussed the interventions utility in supporting SDM conversations (legitimation).

*I think it just gives a structure*,* or clarity to patients*,* which is often needed when we give them a barrage of information … What they’ll come away with is probably quite limited*,* whereas having a resource where they can refer back to that is useful*,* both for us and them. [P0022*,* physiotherapist]*

Commitment to operationalise the intervention was supported by availability of the intervention on paper (utilised by patients and physiotherapists) and paper resources outlining trial procedures (utilised by physiotherapists). However, some patients reported utility of the online version in supporting use of the intervention at ‘unexpected times’, demonstrating ongoing commitment to engage with the material.

One participant reported a reduced commitment from their physiotherapist to drive the SDM intervention within the consultation. As a result, they sought support to operationalise the intervention with another therapist not participating in the trial. Some physiotherapists also reported reduced commitment from some patients where the decision about treatment had already been made with the orthopaedic consultant (discussed above, capacity).

Physiotherapists reported they would engage with the intervention with future patients in varying amounts, from discussing the material and quoting the statistical data to providing patients with the intervention and allowing time before discussing it at a subsequent appointment. One physiotherapist suggested use of a QR code to support engagement in practice and to save paper NHS resource. Another suggested use of a reminder in electronic patient records detailing the core components to cover as part of the SDM consultation to support clinicians to remain engaged with intervention delivery.

### Theme 4c: contribution (collective action)

The work involved in carrying out the intervention was described as non-burdensome by both patients and clinicians. Patients described their ability to read and interpret the information and translate this into meaningful knowledge despite their self-assessed lack of prior medical knowledge (discussed in Topic 1, acceptability and effectiveness). Physiotherapists described the utility of the intervention training session in clearly defining the skills needed to deliver the intervention. There was evidence of Physiotherapists altering their approach to consultations supported by the SDM intervention, to tailor language and discussions to individual patient needs and expectations.

### Theme 4d: contribution (reflexive monitoring)

The translation of knowledge about the intervention’s effectiveness to reconfigure action was identified from both a patient and physiotherapists’ perspective. Participants acknowledged the positive effects of the SDM intervention on SDM process which appeared to support participants (particularly physiotherapists) to evaluate and consider its future use. This was expressed by patients who reported they would recommend the intervention to future patients following diagnosis of an ACL rupture and by clinicians who had utilised the intervention with patients outside the trial. Understanding the impact of the SDM intervention on decision-making and awareness of the final decision made about treatment seemed important to physiotherapists and may impact their continued contribution to operationalise the intervention. Understanding satisfaction with treatment decision making (and decisional regret) was also highlighted by one patient to support their appraisal of engaging with SDM on outcomes of treatment.

Some patients also described continually revisiting the intervention (discussed above, potential). A positive feedback loop was observed where each time they engaged with it, they had increased knowledge about their injury and treatment options and gained confidence to consider non-surgical intervention. This subsequently appeared to reconfigure their action to support their engagement with SDM.

Physiotherapists further described professional skill development as a result of participating in the trial and compatibility of the intervention to be reconfigured to meet the demands of each patient. Both factors improved their views on future implementation in usual care.

### Overview

An overview of the implementation factors identified through ENPT as barriers and enablers to future normalisation are summarised in Table 2.

**Table 2:** Barriers and enablers to implementation and normalisation mapped to ENPT

	Potential	Capacity	Capability	Contribution
Enablers	Individual intention (patient and physiotherapist) to enact the intervention. Translation of beliefs into action.	Intent to incorporate intervention in clinical practice. Timing of intervention delivery allowing for introduction of material, independent & collaborative engagement. Time given between interaction points. Setting expectations and supporting change in social roles and norms around decision-making in healthcare. Trial material (training, website, summary documents and fidelity checklist) facilitated delivery and enactment. Culture of cooperation amongst physiotherapists.	The SDM intervention supports interactional work, offers structure and flows with an initial physiotherapy consultation. Adaptability of the SDM intervention to individual patients. Potentially context independent – demonstration of success outside trial procedures and compatibility with other healthcare settings.	Patients and physiotherapists understand the interventions' purpose, it’s difference to usual care and other resources (coherence). Paper and online resources supported commitment (cognitive participation). Non-burdensome to patients and physiotherapists with use of accessible language (collective action). Positive feedback loop – ongoing engagement (reflexive monitoring). Supports physiotherapists’ professional skill development (reflexive monitoring).
Barriers	Communication of equipoise and context created for intervention delivery crucial to engagement. Shared commitment owned by the orthopaedic team which precedes intervention delivery.	Uncertainty of the culture of cooperation across the pathway. Perceived lack of intent of the other party and their priorities (patient of physiotherapist or vice versa). Uncertainty with decision-making and patient motivation to be involved.	Confidence and competence of less experienced physiotherapists to engage with the intervention and SDM (those not static in a musculoskeletal/orthopedic environment).	Lack of understanding of the interventions impact on the final decision about treatment, patient’s satisfaction with decision and decisional regret (reflexive monitoring). Varying commitment to operationalise the intervention (cognitive participation).

Implementation factors identified in Table 2 were further used to refine the IRLM, considering strategies of action not previously identified to support implementation in a future trial/clinical practice.

## Discussion

The results of this study explored implementation through the lens of ENPT. Barriers and enablers to implementation and future normalisation were identified to support future research and operationalisation in clinical practice.

Physiotherapists viewed the initial appointment in orthopaedics (where the intervention was delivered in the feasibility study to most participants) as the most beneficial time for patients to first interact with the SDM intervention. They reflected that this would maximise the potential of normalisation in the pathway and align with social roles and norms within the healthcare system. This was mirrored by patients who perceived provision of the material at this appointment to offer greatest benefit in engaging with SDM. There were three key interaction points to delivery of the SDM intervention: (1) provision of the material, (2) independent engagement, (3) discussion with the physiotherapist. All three interaction points supported engagement and operationalisation of the intervention in practice with individual intent displayed by patients and physiotherapists. Both patients and physiotherapists identified the intervention as novel and reflected positively on the gap it filled within the current care pathway. It has previously been acknowledged that SDM is more likely if decision support tools have been developed for use in face-to-face encounters, as those designed for use in isolation by the patient outside of clinical practice may not directly influence SDM [16]. Physiotherapy participants in this study praised the intervention for its utility in informing and empowering patients following independent engagement. Its adaptability with supporting SDM during face-to-face and telephone consultations was also reflected upon positively. The combination of both components supporting independent and collaborative engagement is therefore likely to support future implementation and sustainment within practice.

Physiotherapists identified the responsibility for creating and sustaining shared commitment to be owned by the orthopaedic team. This shared commitment across the pathway constructs collective readiness and provides context for the delivery of and engagement with the SDM intervention, the ability to enact it and respond to changes brought about by its operationalisation. Whilst physiotherapists sensed patients expected them to engage with the SDM intervention and be involved in the decision-making process (with expectations set by the intervention), they also self-identified that it was within their role to do so.

There was evidence that the intervention altered the social roles and norms of participants within the pathway. Decision-making can be viewed as a spectrum from informed choice (where the decision is patient led) to paternalism (where the decision is clinician led), with SDM sitting in-between (responsibility for deciding on treatment is shared between the clinician and patient) [17]. The intervention appeared to challenge patients’ perception of social norms and roles and whilst uncertainty was experienced by some, others reflected positively on the intervention enabling them to take a pragmatic approach to decision-making. The intervention supported them to understand outcomes in relation to the current literature, weigh up the pros and cons of treatment and confirm or consider challenging the recommendation offered by the surgeon to proceed with ACLR.

A key component of SDM is the context in which it is presented. It has previously been highlighted that many patients do not expect to be involved in decision-making [16]. Some, for example, may have views of healthcare decision-making in line with a paternalistic approach (identified in this study with patients referring to the surgeon’s decision as final, trusting their judgement as the expert) and some may already align with the SDM model or welcome an intervention supporting this. Previous research has demonstrated that patients who are initially hesitant to participate in decision-making often change their mind after presentation of their options [18]. To support successful implementation in future practice, evidence from this study highlights the importance of clinicians understanding a patient’s motivation to be involved in decision-making (to what extent and how this may change), their expectations of clinicians and what support they may require to enact their preferences and beliefs. Understanding congruency of the SDM intervention with patients pre-conceived ideas of decision-making in healthcare and subsequently how these may change following engagement with the intervention is an important element to consider in a future trial.

A shared commitment to operationalising the intervention across the pathway is another important reflection from this study which is likely to impact future normalisation of the intervention. It is also important to acknowledge that SDM practices may be governed by wider contextual factors that may not have been identified in this study. For example, organisational and system-level factors, such as organisational priorities and financial incentives/allocations of service funding. This may also be an important element to consider in a future trial to support a greater understanding of the intervention’s implementation and translation to effective SDM processes.

Whilst the utility of an SDM intervention is explored in this study, understanding who the intervention may not be appropriate for, may also warrant future exploration as this is likely to support or inhibit sustainment of the intervention in practice and ensure it is enacted with all those who stand to benefit from it. Clinicians identified that in cases where patients had been listed for surgery, this may not allow for full engagement with SDM (as it is perceived a decision has already been made). Therefore, delaying formally listing a patient for ACLR may be necessary to provide greater opportunity for patients to accept their role in decision-making and thus engage with the SDM intervention. However, this may not be appropriate for all and not listing may cause unnecessary delay in treatment delivery. Further, the communication of equipoise around treatment options and cooperation with the intervention was questioned by some physiotherapists who described the importance of a consistent message across the pathway. A lack of equipoise in both patients and clinicians was highlighted in the recruitment of ACL SNNAP (UK pragmatic RCT comparing surgery and rehabilitation following ACL rupture) with 57% declining to participate in the trial due to a preference for surgery and 24% due to a preference for rehabilitation [4]. Creating an awareness of equipoise has previously been described as the first and most important step in SDM [19, 20]. This culture of cooperation, or lack of, is therefore an important consideration. Future research utilising the Quintet Recruitment Intervention and consultation observations may support understanding of recruitment and cooperation with the intervention at the point of its provision in orthopaedics.

Physiotherapists also identified a lack of follow-up to ascertain the final decision made by patient participants as a potential barrier to sustainment in practice, as without this, clinicians are unaware of the intervention’s utility on decision-making. Patients further identified the desire to understand patients’ satisfaction with treatment and decisional regret to support their appraisal of the intervention. A 2016 systematic review highlighted the paucity of high-quality evidence reporting patient satisfaction with ACL treatment [21]. A 2016 Swedish study reported only 19% of participants (*n* = 177) to be ‘happy’ with their knee at a minimum of 12-months post ACLR in response to the question “If you were to spend the rest of your life with your knee function just the way it has been in the last week, would you feel…”. A further 25% reported feeling ‘satisfied’ and so these scores were combined to determine that 44% of patients were satisfied at an average of 3-years following surgery (range 1–7 years) [22]. In contrast, a much higher rate of satisfaction following ACLR (with hamstring graft) was reported in Denmark at 82% at a median of 3.6 years (IQR 1.5) post-ACLR [23]. Relevant to the UK context, patient satisfaction with treatment (surgery or rehabilitation) was reported at 18-months in ACL SNNAP by asking patients to reflect on the nature of their problems in comparison with pre-treatment [4]. 83% of participants in the surgical group reported their knee to be better than pre-treatment with 80% stating they would choose surgical treatment again. In comparison with non-surgical treatment where 68% of participants reported their knee to be better than pre-treatment and 61% would opt for the same treatment. 18% stated they would not choose rehabilitation as a treatment again, compared to 5% in the surgical group. Communicating this information and providing greater detail on satisfaction and decision regret may drive future operationalisation of the intervention in practice, as patients appraise the impact of SDM on treatment outcomes. Use of the SDM intervention may subsequently improve satisfaction and could therefore be an area for future exploration.

A 2012 systematic review explored effectiveness of SDM interventions on the adoption of SDM in clinical practice (assessed from patients’ perspective) [24]. A total of 21 studies comparing 25 interventions were included in the review. The interventions deemed successful combined training of clinicians responsible for SDM with use of a patient-mediated intervention (such as a patient decision aid) [25–27]. Whilst the physiotherapist training in the present feasibility study was not considered part of the intervention, this may be an important component for future sustainment of the intervention and translation of its use into effective SDM practices. Physiotherapists involved in the study identified the lack of prior formal training in SDM and recognised the role of participating in this study in their skill development (discussed in relation to delivery of SDM with ACL patients and wider concepts of SDM utilised with patients with other MSK/orthopaedic conditions). Physiotherapists reflected on the interventions utility in supporting them to deliver SDM practices which is likely to support sustainment of the intervention within usual care. Lack of clinician training has previously been identified as a barrier to implementing SDM and is therefore an important element which may influence normalisation of the intervention in usual practice [28].

### Future directions

Investigating clinician equipoise in the management of ACL ruptures appears warranted. Evidence from this study demonstrated that orthopaedic colleagues were less likely to communicate equipoise for ACL injury treatment (as reported by patients). This subsequently resulted in reduced engagement with SDM, particularly for those with views aligning to paternalistic models of decision-making. Exploring the wider landscape and different clinicians’ perspectives may support further understanding of the context for SDM with patients following an ACL rupture. Exploring clinician equipoise may also identify clinician training needs and targets for future implementation strategies and/or interventions. Understanding patients’ perceptions of SDM in ACL injury management in different healthcare contexts may also be beneficial. This would support understanding of common issues across different healthcare models (e.g. private and government funded) and where initial consultations are conducted with clinicians other than surgeon’s (e.g. physiotherapists working in advanced practice roles). Exploring SDM in other orthopaedic conditions, where surgery is an available treatment option, may further support identification of barriers that are considered specific to ACL injury management and those which may be more generalisable across orthopaedic pathways. It is important that future research regarding SDM in ACL injury management, and likely any orthopaedic condition, engages with surgeons as a key agent in conveying equipoise creating the opportunity for SDM. Observation of clinical consultations where treatment options are discussed may support greater understanding of this.

### Strengths & limitations

Strengths of this study are that views of both patients and physiotherapists were explored to understand implementation from both perspectives. Analysis of interview data utilised ENPT to support understanding of implementation which allowed for deeper exploration of factors that may support or inhibit routine operationalisation of the intervention in usual practice.

The IRLM was used to support planning of study procedures and data form this study has been used to refine the IRLM, considering strategies of action not previously identified to support implementation in a future trial/clinical practice.

This was a single site study and so implementation factors at other NHS trusts may differ and warrant further exploration. Other factors affecting implementation and normalisation may exist that were not identified in this study. Overall generalisability of the intervention to other systems is therefore limited.

## Conclusion

Implementation factors, explored through the lens of ENPT, have supported understanding of future normalisation of the intervention in clinical practice and areas of focus ahead of future use in practice and/or further research.

## Electronic Supplementary Material

Below is the link to the electronic supplementary material.

Supplementary Material 1

Supplementary Material 2

Supplementary Material 3

Supplementary Material 4

Supplementary Material 5

## Data Availability

All data generated or analysed during this study are included in this published article [and its supplementary information files].

## Declarations

### Authors’ information

Nil.
