# Supplementary figures and images for: Exploring implementation of a shared decision-making intervention for patients following an Anterior Cruciate Ligament rupture: a qualitative investigation
Source: BMC Med Inform Decis Mak. 2026 Mar 17;26:142. doi: 10.1186/s12911-026-03430-3 (PMC13107614; doi:10.1186/s12911-026-03430-3)

## Supplementary File 1 – Implementation Research Logic Model

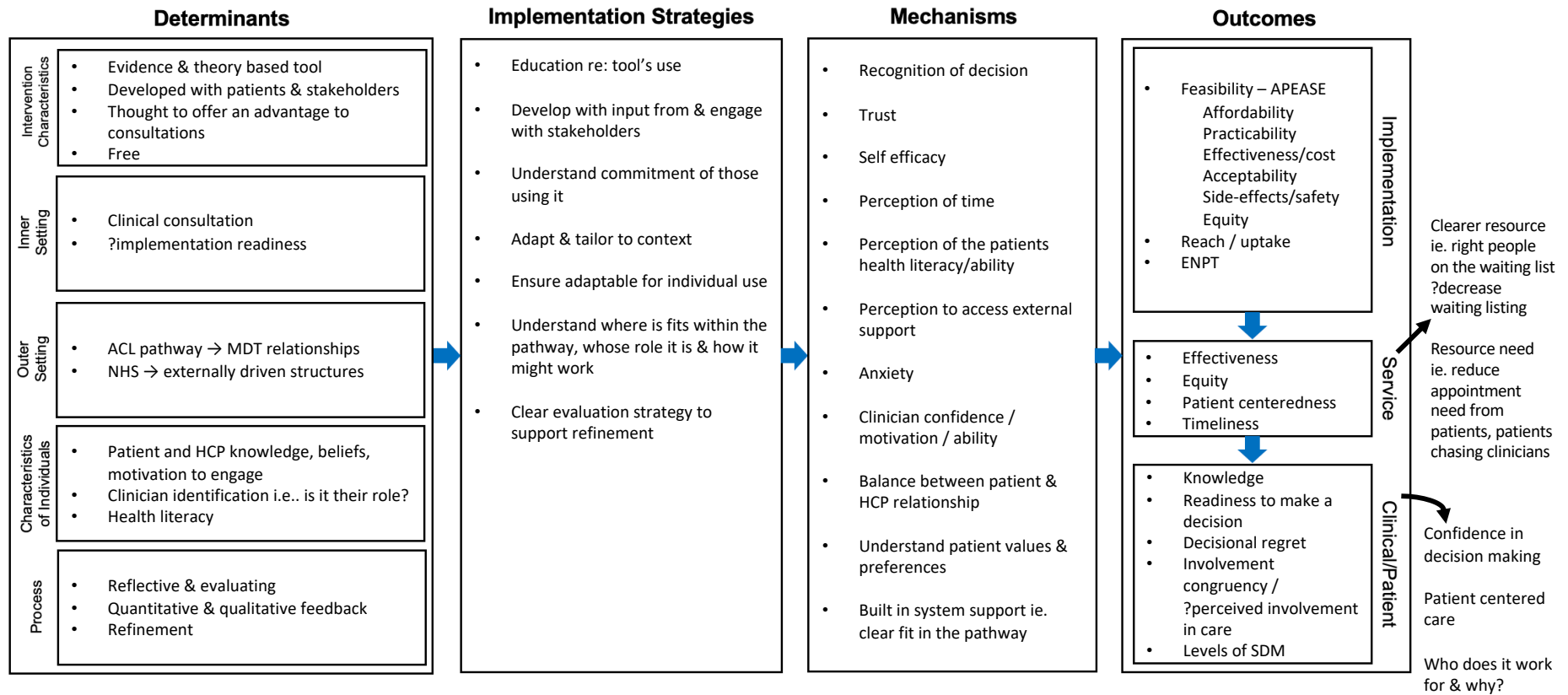

Supplement: Supplementary file 1 — Supplementary Material 1 [file 12911_2026_3430_MOESM1_ESM.pdf]
