# Supplementary material for: Exploring implementation of a shared decision-making intervention for patients following an Anterior Cruciate Ligament rupture: a qualitative investigation
Source: BMC Med Inform Decis Mak. 2026 Mar 17;26:142. doi: 10.1186/s12911-026-03430-3 (PMC13107614; doi:10.1186/s12911-026-03430-3)
Supplement: Supplementary file 5 — Supplementary Material 5 [file 12911_2026_3430_MOESM5_ESM.pdf]

The University of Nottingham
UNITED KINGDOM · CHINA · MALAYSIA

NHS
University Hospitals of Derby and Burton
NHS Foundation Trust

Interview Topic Guide – Participant / Clinician Interview

(delete as appropriate for interviewee)

Version 1.0 – 23/10/2023

Introduction and Background

- Thank you for agreeing to take part in the study to discuss use of the shared decision making tools
- Tell me about your experience of using the tools

Possible prompts:

- Helpfulness of tools
- Length / readability / layout

Topic 1: Acceptability

Possible prompts:

- Acceptability of using the tools
- Thoughts on acceptability of use in practice
- Where they helpful in decision making about treatment
- Format – paper vs online

Topic 2: Implementation Factors

Possible prompts:

- Where the tools burdensome to use?
- For participants only
    - Where you happy using the tools with the physiotherapist? Whose role do you think it should be?
    - Did you feel the tools allowed for a consultation and discussion specific to you and your goals
- For clinicians only
    - Was the education you received in training sufficient to use the tools?
    - How you do envision the tools being used in practice?
    - Were the tools compatible with current practice?
    - Did you feel able to use the tools with every participants e.g were you able to adapt/tailor consultations using them
    - Did you feel like it was your role to deliver the tools?
    - How do you think we should be evaluating the tools?

[Clinicians only] Topic 3: Contamination

Possible prompts:

- Having used the tools – how do you think it's impacted your practice in consultations where you have not used the tools (for ACL patients and other MSK conditions)

Close

- Is there anything you feel could be changed about the SDM tools
- Anything further you would like to mention/discuss
- Thank you for taking the time to discuss your experience
